# An Actualisation of the Study of Morbid Inheritance

**Published:** 1909-05-08

**Authors:** 


### AN ACTUALISATION OF THE STUDY OF MORBID INHERITANCE.

DESPITE the prominent position in biology earned by British workers little serious attention in this country has been given the subject of hereditary transmission of defect and of proclivity to disease. No doubt there are some good reasons for this. The years since Darwin came to his own have witnessed also great achievements of bacteriology in the revelation, and in many directions in the combating, of hitherto unknown *materies morbi*. It is not strange that a practically minded people should have been much impressed and taken up with the new methods of investigation and their hygienic and therapeutic possibilities. It may be, however, that the present-day conception of medical research has become so much influenced by the great use made in late years of the microscope and of physicists' apparatus generally, that we have come to think more of the photographer's fingers than of the philosopher's brain, forgetting that in science there is nothing common or unclean, and that simple facts requiring for their collection and appreciation rather temperament than technique may build none the less an imposing edifice of knowledge. At first sight data concerning morbid inheritance, as at present ascertainable, seem greatly lacking in that precision necessary to base trustworthy conclusions upon them. As all medical men are aware, diagnosis itself is often far from exact. In only a small proportion of cases may there be opportunity of establishing the nature of the disease by means of purely objective evidence. Even lay statements, with all their glaring imperfections, have to be relied upon largely. Yet, allowing for all this, there may be much of value to be learned, much of a significance not to be destroyed by fine-drawn deductions from the results of laboratory experiment. If, to take a simple instance, it be the case (as one observer maintains) that cancer patients give a family history of consumption disproportionately often, then this is a fact worth careful note. It is evident that if a mere counting of heads like this can reveal nothing, there must be plenty of scope in this branch of research for advanced statistical methods; and in this country we are fortunate in possessing in Professor Karl Pearson a mathematician whose methods, a development of those of Laplace and Gauss, are conceded to have broken much fresh ground in the direction of measuring the correlation of organic characters, particularly in connection with the subject of inheritance.

A hearty welcome may therefore be extended to a publication* hailing from the Eugenics laboratory under Professor Pearson's direction, and containing much bibliographical and original research by medical workers on various diseases and abnormalities, especially as in the foreword by Mr. Francis Galton, and in the preface, a non-controversial note is sounded, and the intention repeatedly expressed to draw no premature inferences from the material thus garnered. Save for one small section the work deals exclusively with disease and malformation. Dr. Bulloch extracts from the haystack of modern medical literature such needles as the bibliographies of heredity in diabetes insipidus and chronic hereditary trophœdema. With regard to the first-named

* "The Treasury of Human Inheritance." Parts I. and II. Pp. 38+x., with illustrations. University of London, Francis Galton Laboratory for National Eugenics. (London: Dulau and Co. 1909. Price 14s.)

disease, a point may be mentioned which holds good of several papers in the series. Where heredity is not an invariable characteristic of the affection studied, the cases given should be supplemented, at any rate before conclusions come to be drawn, by others in which no inheritance is observable. Drs. Lewis and Embleton contribute carefully studied memoirs on split-foot, polydactylism, and brachydactylism, illustrated by radiographs and photographs, some of these reproduced from an article by these authors recently appearing in *Biometrika*. There is a good bibliography of the hereditary aspect of deaf-mutism, and Dr. Jobson Horne writes a general account of the disease. Dr. Rivers gives some pedigrees of consumptives, in the case of two of which the distribution of consumption in the family seemed to be roughly parallel with that of ichthyosis. All of the foregoing cases are recorded in detail in the letterpress, and graphically by means of excellently devised charts.

The co-ordination of all this multifarious detail, or rather of such of it as admits of the process, must needs be a task of great difficulty and uncertainty regarding result. It is legitimate, however, to record the impression gained that the study of morbid heredity is probably a much more complicated thing than merely looking for Mendelian characteristics, a subject which attracted much discussion at the recent debate at a meeting of the Royal Society of Medicine. It has been said that in this country morbid heredity has had little attention, and the same holds good of anywhere except France and perhaps Germany. What little we may guess as to the actual genealogical distribution of disease we owe to writers like Morel, Moreau, Delage, and Féré. And the tenor of their opinion is that disease may descend from generation to generation not *per se*, but in divers forms and manifestations. "Hérédité dissemblable" may be an instance of the superficial generalisation to which French medicine is rather prone, or it may not. The prevalence of the conception of the "degenerate" among followers of some of the writers named may have as little importance as in this country, where Lombroso's ideas are a chopping block for anthropologist, sociologist, and physician alike, might naturally be presumed. But certainly in many of the families recorded in this thesaurus there are more than the one abnormality to be noted. Idiocy, insanity, and blindness are seen among deaf mutes, epilepsy in the subjects of chronic trophœdema. The family of Zerah Colburn, the calculating boy, figures amongst those affected with polydactylism. A pedigree of diabetes insipidus shows two individuals with physical abnormalities and two who were idiots. This may show the difficulty of running the measuring rule over such material, but it will also strengthen the hand of the advocate of eugenics against those who in effect maintain that hygiene is the only means of racial improvement.

